# Whole-liver enhanced CT radiomics analysis to predict metachronous liver metastases after rectal cancer surgery

**DOI:** 10.1186/s40644-022-00485-z

**Published:** 2022-09-11

**Authors:** Meng Liang, Xiaohong Ma, Leyao Wang, Dengfeng Li, Sicong Wang, Hongmei Zhang, Xinming Zhao

**Affiliations:** 1 Department of Diagnostic Radiology, National Cancer Center/National Clinical Research Center for Cancer/Cancer Hospital, Chinese Academy of Medical Sciences and Peking Union Medical College, Beijing, 100021 People’s Republic of China; 2 GE Healthcare (China), Beijing, 100176 People’s Republic of China

**Keywords:** Rectal cancer, Radiomics, Liver metastases, Computed tomography

## Abstract

**Background:**

To develop a radiomics model based on pretreatment whole-liver portal venous phase (PVP) contrast-enhanced CT (CE-CT) images for predicting metachronous liver metastases (MLM) within 24 months after rectal cancer (RC) surgery.

**Methods:**

This study retrospectively analyzed 112 RC patients without preoperative liver metastases who underwent rectal surgery between January 2015 and December 2017 at our institution. Volume of interest (VOI) segmentation of the whole-liver was performed on the PVP CE-CT images. All 1316 radiomics features were extracted automatically. The maximum-relevance and minimum-redundancy and least absolute shrinkage and selection operator methods were used for features selection and radiomics signature constructing. Three models based on radiomics features (radiomics model), clinical features (clinical model), and radiomics combined with clinical features (combined model) were built by multivariable logistic regression analysis. Receiver operating characteristic (ROC) curves were used to assess the diagnostic performance of models, and calibration curve and the decision curve analysis were performed to evaluate the clinical application value.

**Results:**

In total, 52 patients in the MLM group and 60 patients in the non-MLM group were enrolled in this study. The radscore was built using 16 selected features and the corresponding coefficients. Both the radiomics model and the combined model showed higher diagnostic performance than clinical model (AUCs of training set: radiomics model 0.84 (95% CI, 0.76–0.93), clinical model 0.65 (95% CI, 0.55–0.75), combined model 0.85 (95% CI, 0.77–0.94); AUCs of validation set: radiomics model 0.84 (95% CI, 0.70–0.98), clinical model 0.58 (95% CI, 0.40–0.76), combined model 0.85 (95% CI, 0.71–0.99)). The calibration curves showed great consistency between the predicted value and actual event probability. The DCA showed that both the radiomics and combined models could add a net benefit on a large scale.

**Conclusions:**

The radiomics model based on preoperative whole-liver PVP CE-CT could predict MLM within 24 months after RC surgery. Clinical features could not significantly improve the prediction efficiency of the radiomics model.

**Supplementary Information:**

The online version contains supplementary material available at 10.1186/s40644-022-00485-z.

## Introduction

Due to its anatomical condition in relation to its portal circulation, colorectal cancer (CRC) cells are most likely to metastasize to the liver [1, 2]. The high incidence of liver metastases (LM) is the major factor leading poor prognoses in patients with CRC [1–3]. In recent years, many studies have focused on the effect of metastatic target organs on tumor cell colonization [4]. A variety of liver-based factors that influence LM of CRC have been confirmed. Hepatitis B virus positive has been considered to be an independent protective factor influencing the occurrence of LM in CRC [5–7], which shows higher progression-free survival and overall survival rates in the infection group [8]. In addition, the effect of fatty liver on LM of CRC has been widely concerned in recent years [9], but no unified conclusion has been reached. Some studies believed that the fatty liver microenvironment was conducive to tumor progression [10], while others thought that moderate fat changes could inhibit the growth of LM [11]. This finding may be attributed to the influence of liver microenvironment changes induced by diffuse liver injury on the planting of metastatic cells in the liver. The liver shows complex hemodynamic changes owing to its dual blood supply, and the liver blood perfusion index of patients with colorectal LM has been reported to be significantly higher than that of patients without LM and healthy controls [12]. Therefore, in addition to macroscopic diffuse liver lesions, changes in the liver microenvironment also play important roles in LM progression.

Tumor cells colonize the liver through a series of complex processes and are limited by oxygen diffusion [13]. Approximately 24–81% of CRC patients were reported to have micrometastases in liver surgical resection specimens [14], which were considered invisible to the naked eye and imaging examinations before surgery and confirmed by postoperative pathological diagnosis. In cases showing occult metastasis, these micrometastases or occult metastases can cause changes in the liver microenvironment, making it more heterogeneous [15, 16]. Metachronous liver metastases (MLM) occurring shortly after the initial diagnosis often develop from these occult metastases. Therefore, analysis of the liver microenvironment is valuable for predicting the occurrence of MLM.

Portal venous phase (PVP) contrast-enhanced CT (CE-CT) is the most common method used for the diagnosis of LM, but because of its soft tissue resolution, it generally has low sensitivity for detecting lesions with a diameter of < 1 cm [17]. Although some studies have highlighted the advantages of MRI, especially DWI and application of gadoxetic acid, in the diagnosis of LM, and FDG PET/CT may also facilitate LM diagnosis [17, 18], these examination methods are not as widely used as enhanced CT in clinical application. Therefore, the ideal approach is to use standard imaging techniques, such as classical PVP CE-CT, to develop reproducible and available prediction models to determine the heterogeneity of the liver microenvironment in the patients with occult LM or MLM in the next 24 months. This approach will highlight the patients at a high risk of MLM, guiding the clinical strategy and yielding a reasonable individualized treatment plan. In this regard, radiomics has shown an important value in the heterogeneity analysis of tissues and lesions [19].

The aim of our study was to build and verify a radiomics model based on pretreatment whole-liver PVP CE-CT images to predict MLM within 24 months after rectal cancer (RC) surgery.

## Materials and methods

### Patients

Patients with RC who underwent surgery between January 2015 and December 2017 at our institution were screened in this retrospective study through reviewing their imaging, clinical, and pathological data and information regarding postoperative treatment and follow-up evaluations. The ethics committee of our institution approved this study and waived informed consent.

The inclusion criteria included: (1) patients with rectal adenocarcinoma who underwent RC surgery at our institution and did not received antitumor treatment before surgery; (2) abdominal PVP CE-CT performed at our institution within 1 month before surgery; (3) initial stage was M0 with diagnosis of RC, and first metastasis appearing in the liver during follow-up; (4) no history of malignancy of other organs; (5) no history of liver disease treatment (except for hepatitis); (6) availability of complete follow-up imaging data obtained during regular follow-up at our hospital; and (7) no contraindications for CE-CT examinations.

The exclusion criteria included: (1) rare pathological types of RC (except for rectal adenocarcinoma), (2) distant metastases or undetermined lesions found before the operation, (3) metastases to other organs before LM, (4) lack of preoperative abdominal PVP CE-CT images, (5) obvious motion artifacts or metal artifacts in CT images, (6) multiple or diffuse benign lesions (such as cysts and hemangiomas) in the liver affecting image segmentation of liver parenchyma; and (7) incomplete clinical indicator or follow-up data.

Two groups were analyzed in this study: (1) the MLM group, defined as RC patients whose metastasis stage was M0 at initial diagnosis, but LM appeared within 24 months after surgery (no other organ metastases occurred before LM), and (2) the non-MLM group, defined as RC patients who showed no metastatic diseases preoperative and within 24 months postoperative images.

### Acquisition and scanning parameters for CT imaging

The Toshiba Aquilion, GE Optima CT660, GE Discovery 750 HD, and GE Lightspeed VCT 64-slice spiral CT scanners were used for examinations. The patients fasted for 4–6 hours before the examinations. The examinations were conducted with the patient in the supine position (examinations involving simultaneous abdominal-pelvic CT scans were conducted with the patient in the prone position and with intestinal preparation), both arms raised. The scanning range included at least the entire abdomen. A non-ionic iodine contrast agent (0.3 mg I/mL, Ultravis, Bayer) was injected into the superficial middle elbow vein by using a high-pressure syringe. The dose of the contrast agent was approximately 100 mL with an injection rate of 3.0 mL/s. All patients underwent abdominal scanning with one breath-hold. PVP CE-CT images were acquired with a delay of approximately 65 s after injection of the contrast agent. The scan protocol was as follows: automatic tube current; tube voltage, 120 kV; rotation speed, 0.5 s/rot; screw pitch, 0.984; and slice thickness and layer spacing of conventional scans, 5 mm.

### Collection of clinical information

Clinical information of enrolled RC patients regarding sex, age, pathological primary tumor (pT) stage, pathological regional lymph node (pN) stage, hepatitis infection, carbohydrate antigen 19–9 (CA19–9), and carcinoembryonic antigen (CEA) levels were collected through retrospectively reviewing medical records. We also recorded whether postoperative adjuvant treatment, including chemotherapy and chemoradiotherapy.

### Therapeutic methods and clinical follow-up

Three antitumor protocols in this study: (1) total mesorectal excision (TME) only, (2) TME followed by adjuvant chemotherapy, and (3) TME followed by adjuvant chemoradiotherapy. None of the patients received antitumor treatment before TME.

All patients were followed up once in every 3 months during the first year, every 6 months in second year, and annually thereafter. Patients without metastases were followed up for at least 2 years after RC surgery. Clinical follow-up after surgery was carried out by reviewing medical records, including serology, colonoscopy, and imaging examinations. Follow-up assessments of LM were mainly based on CE-CT scans. Preoperative images of all patients showed no LM. Among cases showing new suspicious liver lesions during follow-up assessments, some lesions were diagnosed by CE-CT scanning, some uncertain lesions required further diagnoses by liver MRI or positron emission tomography/CT, some were confirmed during follow-up, and some were diagnosed by puncture or surgical pathology. The follow-up time was defined as from the first day after RC surgery to the occurrence of LM or the endpoint of follow-up. The final follow-up was conducted on September 30, 2020.

### Image segmentation and radiomics feature extraction

**Fig. 1:**
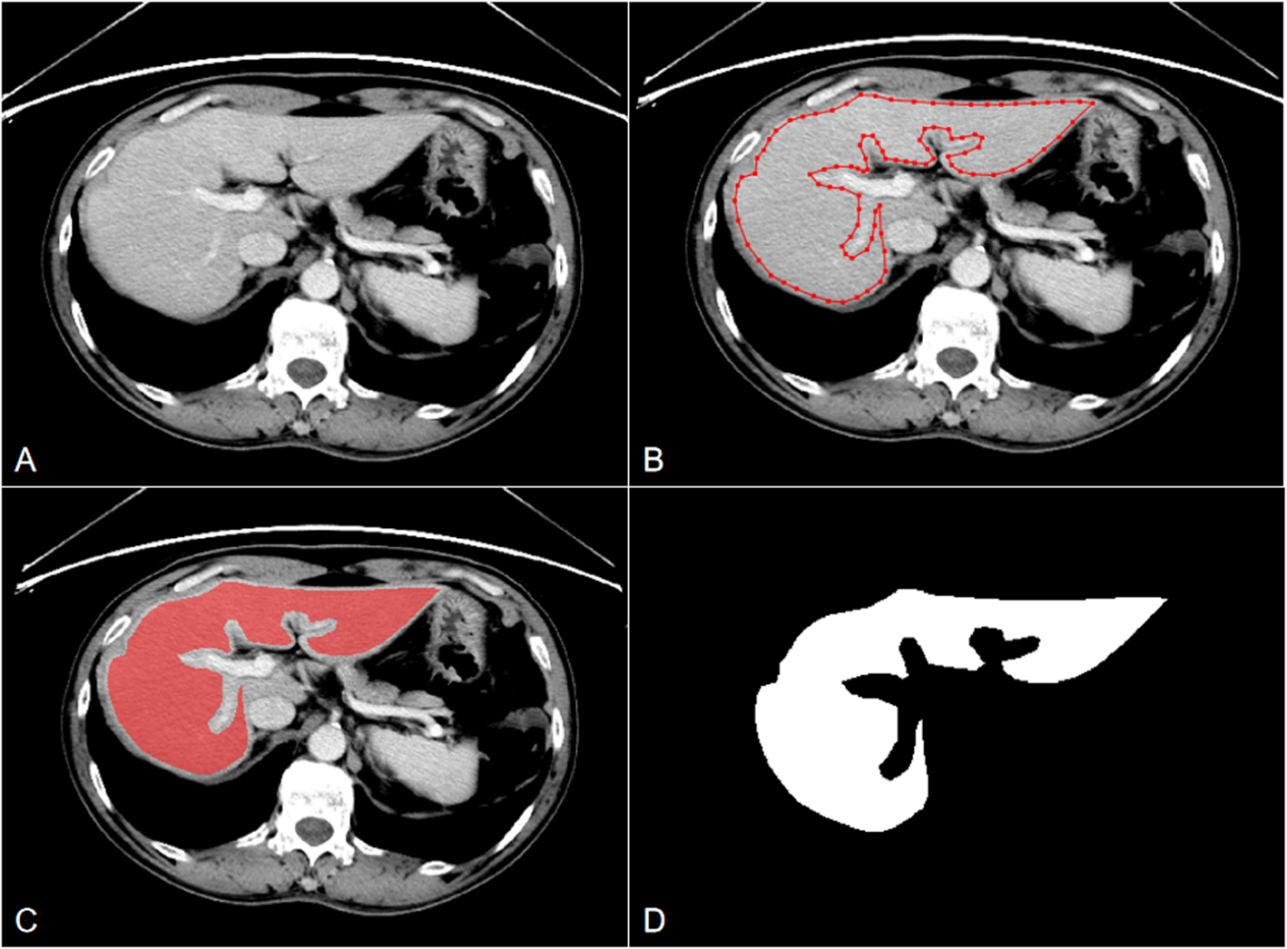
Schematic diagram of manual segmentation of whole-liver VOI. This was a 70-year-old male patient with RC in the MLM group who developed LM on follow-up images in the 13th month after RC surgery. The red outline in the figure shows the scanning-level area of the liver parenchyma without lesions. Whole-liver VOI without lesions was obtained by sketching the liver layer-by-layer, avoiding the edge of the liver, portal vein, inferior vena cava, and hepatic caudate lobe. **A** Original PVP CE-CT image of the liver; **B** manual sketching of one layer; **C** sketching of one layer was completed; and **D** schematic diagram after image segmentation

Volume of interest (VOI) segmentation of the whole-liver was performed on PVP CE-CT images by using the open-source imaging platform ITK-SNAP version 3.8 (www.itksnap.org). Image segmentation was manually performed on all and randomly selected cases by two experienced radiologists with 8 and 6 years of experience in abdominal radiology, respectively. They were both blinded to the clinicopathological information. The liver window was adjusted appropriately to optimize the liver parenchyma display (window width, 200–300 HU; window level, 30–70 HU). The VOIs included the whole-liver parenchyma without lesions on the CT images and were manually delineated layer-by-layer, avoiding the edge of the liver (in order to avoid partial volume effects), visible benign lesions in the liver (including cysts, hemangiomas, and calcifications), the main veins and branches in the liver, and hepatic caudate lobe (due to the unclear boundary between the caudate lobe and inferior vena cava in some patients). A schematic diagram of manual segmentation of whole-liver VOI was shown in Fig. 1.

The segmented images were then imported into Artificial Intelligence kit software (A.K. software; version 3.2.5; GE Healthcare, China). A total of 1316 radiomics features were automatically extracted from the PVP CE-CT images, including 18 first-order histogram features, 14 shape features, 24 Gray-level co-occurrence matrix features, 16 Gray-level size-zone matrix features, 16 Gray-level run-length matrix features, 14 Gray-level dependence matrix features, 5 neighboring gray-tone difference matrix features, 186 Laplacian of Gaussian (LoGsigma = 2.0/3.0) features, 744 wavelet features, and 279 local binary pattern features.

### Radiomics signature construction and validation

All enrolled patients were randomly divided into training and validation sets in a 7:3 ratio. The training set contained 78 patients (36 patients with MLM and 42 patients without MLM), while the validation set contained 34 patients (16 patients with MLM and 18 patients without MLM).

Interclass correlation coefficient (ICC) values of 30 randomly selected samples were used to compare the consistency of manual segmentation between two radiologists. Features with ICC > 0.75 were selected for subsequent analysis to ensure the high value of the radiomics model. The dimensionality reduction process consisted of two steps. Firstly, the max-relevance and min-redundancy (mRMR) method was used to remove redundant features and retain 20 features most related to MLM. Subsequently, the least absolute shrinkage and selection operator (LASSO) with 5-fold cross validation was carried out to obtain the best feature sets for constructing the radiomics signature. The radscore was calculated by summing the selected features and corresponding coefficients. We then compared the radscores between the two groups in the training and validation sets, respectively.

### Construction and validation of prediction models

**Fig. 2:**
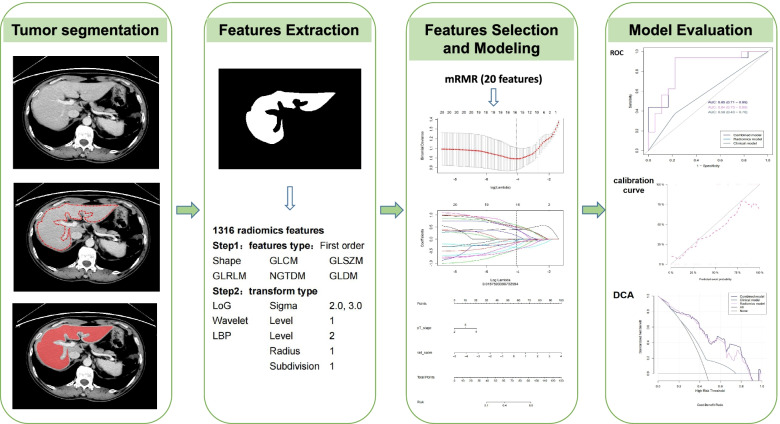
Flow chart describing the workflow for construction and validation of the radiomics model

The clinical model was constructed using the clinical indicators obtained from multivariate analysis. Then, the radscore and significant clinical indicators where *p* < 0.05 in univariate analysis were included in multivariate logistic regression analysis to construct a combined model. A nomogram was created to make the model visible. Receiver operating characteristic (ROC) curves were used to evaluate the effectiveness of three models in predicting MLM, the area under the curve (AUC), specificity, sensitivity, accuracy, negative predictive value, and positive predictive value were calculated. The DeLong test was used to compare the differences in predicting MLM among the three models. The reliability of the nomogram was determined according to its calibration curve, and the goodness-of-fit was evaluated by the Hosmer–Lemeshow test. Finally, decision curve analysis (DCA) was used to calculate the clinical application value of the three models by quantifying the net benefit at different threshold probabilities. All the procedures for building and validating the radiomics models were shown in Fig. 2.

### Statistical analysis

Statistical analyses were performed using R version 3.5.1 software and SPSS version 22.0. The Kolmogorov-Smirnov and Levene tests were used to verify the normal distribution and homoscedasticity of continuous variables. The variables with normal distribution and homogeneous variance were compared by two independent-sample t-tests. Otherwise, the Mann-Whitney U test was used. The chi-square test and Fisher’s exact test were used to compare the categorical variables between groups. Variables showing statistically significant differences in univariate analysis were included for further multivariate logistic regression analysis. A two-tailed *p* < 0.05 was considered statistically significant.

## Results

### Clinical indicators

**Fig. 3:**
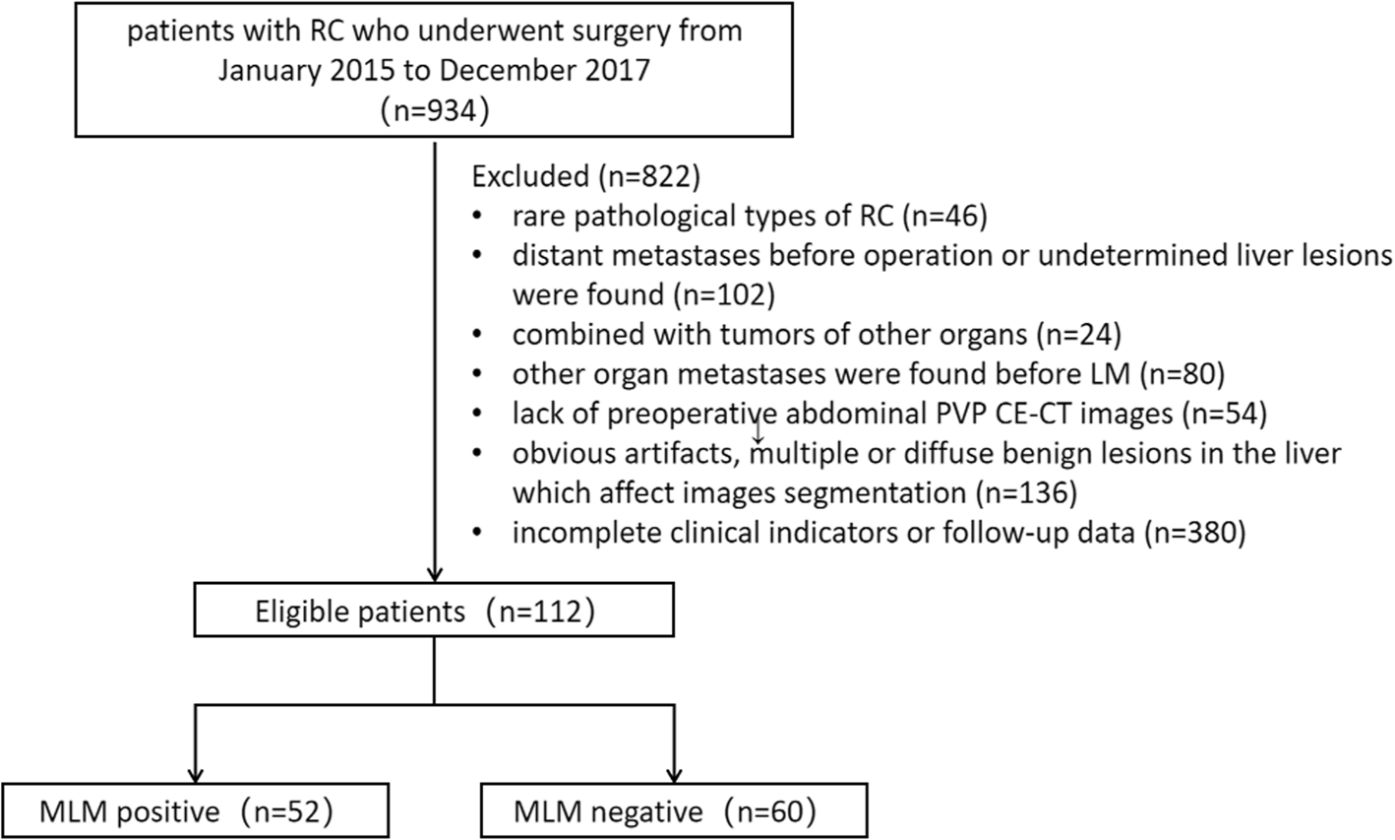
Flowchart of patient enrollment

On the basis of the above inclusion and exclusion criteria, 112 patients were enrolled, including 52 patients in the MLM group and 60 patients in the non-MLM group (flowchart of patient enrollment was shown in Fig. 3). The median follow-up time was 9 months (1–24 months) in the MLM group, and that in the non-MLM group was 42.5 months (36–64 months). In the MLM group, 17 patients underwent TME without adjuvant therapy before LM, 19 patients underwent postoperative adjuvant chemotherapy, and 16 patients underwent postoperative adjuvant chemoradiotherapy. In the non-MLM group, only four patients underwent TME without adjuvant therapy, 20 patients underwent postoperative adjuvant chemotherapy, and 36 patients underwent postoperative adjuvant chemoradiotherapy.

In univariate analysis, risk predictors including sex, pT stage, and CEA level were preliminarily selected (*p =* 0.042, 0.026 and 0.031). After multivariable analysis, pT stage was regarded as an independent risk predictor of MLM (*p* = 0.009, Odds Ratio (OR) = 2.464, 95% confidence interval (CI) 1.252–4.851). The clinical indicators and results of the univariate and multivariable analysis to identify significant clinical factors for MLM were present in Table 1.

**Table 1:** Univariate and Multivariate analysis to identify significant clinical factors for MLM

Clinical Characteristics	Non-MLM Group (*n* = 60)	MLM Group (*n* = 52)	univariate analysis		multivariate analysis	
			Statistics	*p*-value	OR (95% CI)	*p*-value
Age (mean ± SD, years)	56.12 ± 9.84	57.96 ± 10.10	−0.978	0.330		
Sex			4.125	0.042*		
Male	34 (30.4%)	39 (34.8%)				
Female	26 (23.2%)	13 (11.6%)				
pT stage			7.328	0.026*	2.464 (1.252–4.851)	0.009*
pT2	14 (12.5%)	5 (4.4%)				
pT3	39 (34.8%)	32 (28.6%)				
pT4	7 (6.3%)	15 (13.4%)				
pN stage			2.441	0.295		
pN0	8 (7.1%)	5 (4.5%)				
pN1	35 (31.3%)	25 (22.3%)				
pN2	17 (15.2%)	22 (19.6%)				
Hepatitis			1.876	0.171		
Negative	41 (36.6%)	29 (25.9%)				
Positive	19 (17.0%)	23 (20.5%)				
CEA			4.633	0.031*		
Normal (< 5 ng/ml)	43 (38.4%)	27 (24.1%)				
Elevated (≥5 ng/ml)	17 (15.2%)	25 (22.3%)				
CA19–9			1.450	0.229		
Normal (< 37 U/ml)	51 (45.5%)	48 (42.9%)				
Elevated (≥37 U/ml)	9 (8.0%)	4 (3.6%)				

*MLM* metachronous liver metastases; *non-MLM* non-metachronous liver metastases; *SD* standard deviation; *CA19–9* carbohydrate antigen 19–9; *CEA* carcinoembryonic antigen; *OR* Odds Ratio; *CI* confidence interval; * *p* < 0.05

### Radiomics features selection

A total of 1062 features with ICC > 0.75 were obtained for dimensionality reduction. After applying the mRMR and LASSO methods, 16 features were retained. The detailed process of radiomics feature selection using the LASSO method was shown in Supplementary Fig. 1**.** The 16 features and the corresponding coefficients were shown in Supplementary Fig. 2.

### Radiomics signature construction

**Fig. 4:**
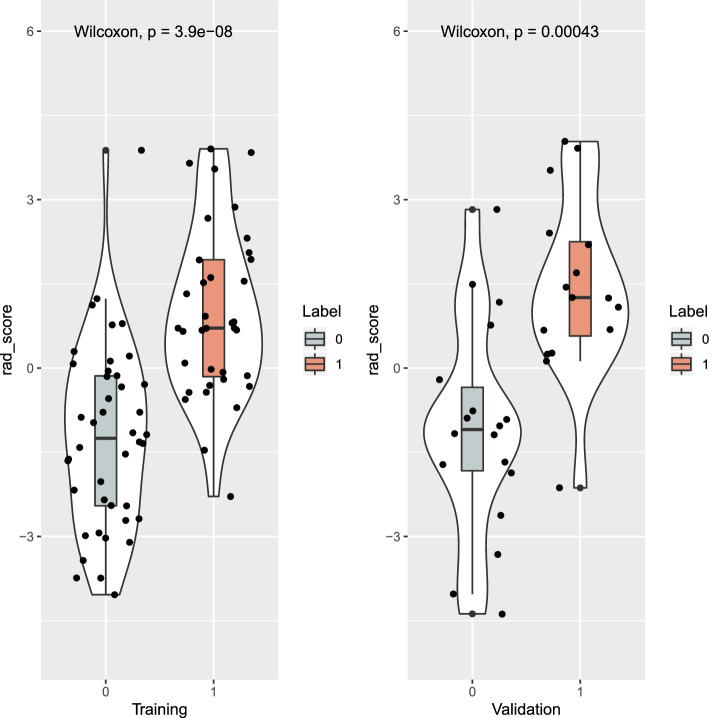
Radscore distribution in the training and validation sets. Boxplot showed that there were significant differences in the radscores between the non-MLM (label 0) and MLM (label 1) groups in the training and validation sets (both *p* < 0.05)

To quantify the radiomics signature constructed by liver PVP CE-CT, we obtained the radscores by a linear weighting of the 16 selected features most relevant to MLM and corresponding coefficients, as shown in the Supplementary formula. In addition, there were significant differences in the radscores between the MLM and non-MLM groups in the training and validation sets (*p* < 0.05). The radscores distribution in two sets was shown in Fig. 4.

### Radiomics nomogram construction and performance evaluation

**Fig. 5:**
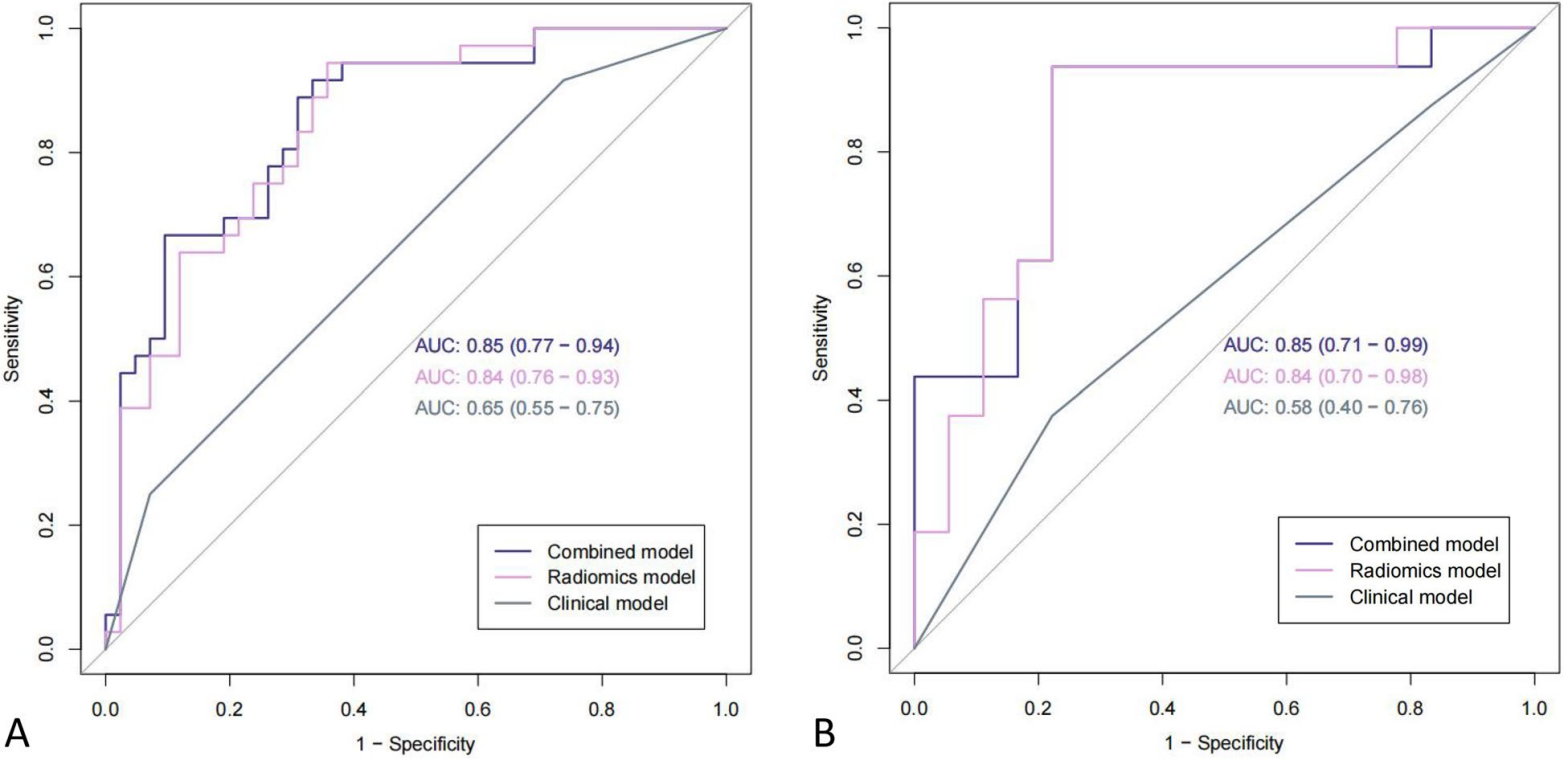
The ROC curves of the clinical model, radiomics model, and combined model to predict the MLM and non-MLM groups in the training set (**A**) and validation set (**B**)

**Fig. 6:**
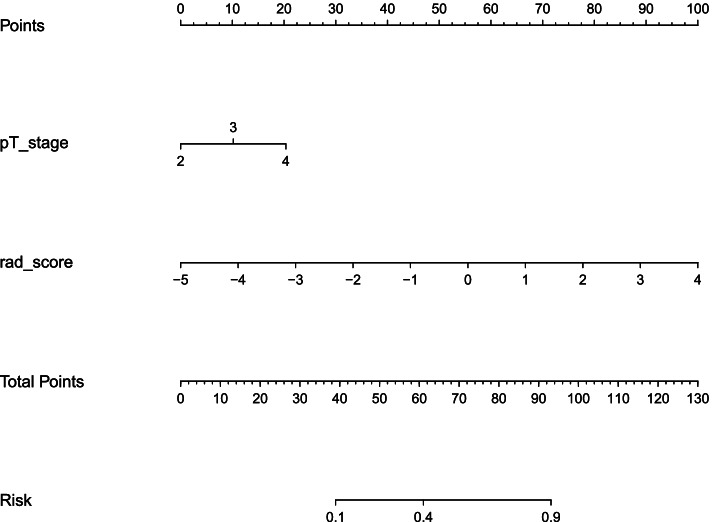
The nomogram for predicting MLM after RC surgery. The nomogram was composed of the radscore and pT stage

In the radiomics model, the AUC in the training set for predicting MLM was 0.84 (95% CI, 0.76–0.93), and the AUC in the validation group was 0.84 (95% CI, 0.70–0.98), indicating a good predictive model. The clinical model was established using the pT stage obtained by multivariate analysis, and the combined model was obtained by combining the radscore and indicator pT stage. Based on the Youden index, the performance parameters were calculated in Table 2, and the ROCs were shown in Fig. 5. To ensure easy use of the model, we presented the results as a nomogram (Fig. 6). The DeLong test confirmed that the radiomics and the combined models were both superior to the clinical model in predicting MLM in the training set (*p* = 0.002 and < 0.001) and the validation set (*p* = 0.012 and 0.003), while the radiomics model and the combined model had similar predictive performance in two sets (*p* = 0.592 and 0.737).

**Table 2:** The predictive performance of the clinical model, the radiomics model, and the combined model

Model	Accuracy	Sensitivity	Specificity	positive predictive value	negative predictive value	AUC (95% CI)
**Radiomics training**	0.782	0.944	0.643	0.694	0.931	0.84 (0.76–0.93)
**Radiomics validation**	0.824	0.938	0.722	0.750	0.929	0.84 (0.70–0.98)
**Clinical training**	0.603	0.568	0.634	0.583	0.619	0.65 (0.55–0.75)
**Clinical validation**	0.500	0.474	0.533	0.563	0.444	0.58 (0.40–0.76)
**Combined training**	0.782	0.702	0.903	0.917	0.667	0.85 (0.77–0.94)
**Combined validation**	0.853	0.789	0.933	0.938	0.778	0.85 (0.71–0.99)

*CI* confidence interval

**Fig. 7:**
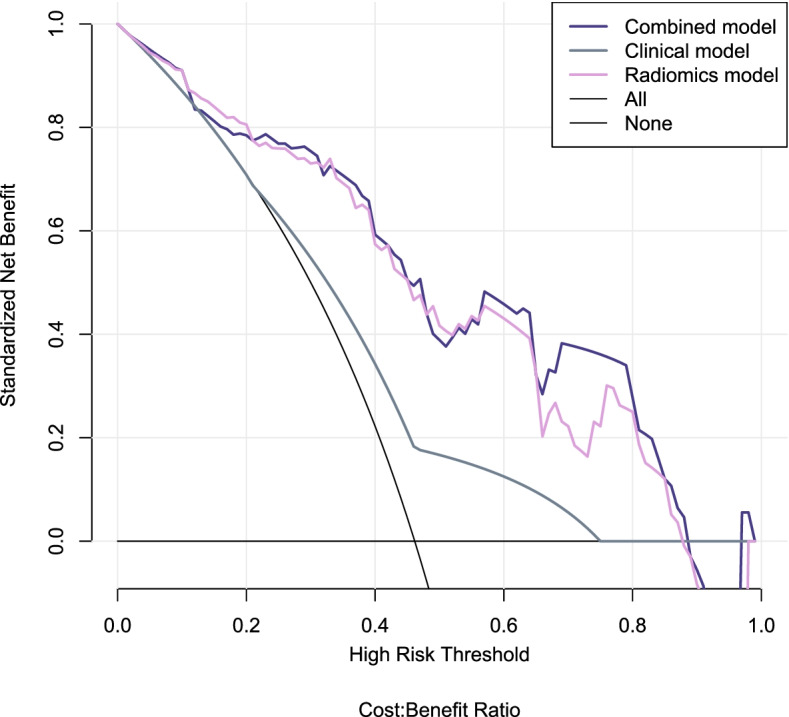
DCA curves of predictive models. The slash curve (All) represents that all MLM status is positive. The horizontal line (None) represents that all MLM status is negative. The three curves (combined model, clinical model, and radiomics model) represent the clinical value for the prediction of MLM. When the threshold probability was 0.12–0.20, the net benefits of radiomics model was greater than that of combined model. When the threshold probability was 0.20–0.90, the net benefit of the combined model was similar or slightly larger than that of the radiomics model, and both were much larger than that of clinical model

In the training and validation sets, the model showed goodness-of-fit in the Hosmer–Lemeshow test (*p* = 0.294 and 0.107). The calibration curve showed that the predicted values of the model in the training set and the validation set were in good agreement with the actual MLM (Supplementary Fig. 3). Finally, DCA demonstrated that either the radiomics or combined model had good clinical application value to predict MLM and added more benefit than using the clinical model only (Fig. 7).

## Discussion

CT and MR radiomics characteristics of CRC have been confirmed in previous studies that have some predictive value for MLM [20–22]. On the basis of a study of primary tumors, we analyzed the effect of the liver microenvironment on MLM. In this study, the radiomics and combined models based on whole-liver PVP CE-CT images showed good efficacy in predicting postoperative MLM in RC patients. The AUC value of the radiomics model and combined model in the validation set were 0.84 (95% CI, 0.70–0.98) and 0.85 (95% CI, 0.71–0.99); the clinical model failed to show better diagnostic ability than the radiomics model. Evaluation of high-risk patients with MLM using preoperative CE-CT images could facilitate early detection of MLM, which plays an important role in individualized treatment strategy and improvement of prognosis of the patients with RC.

In 2007, Ganeshan et al. [23, 24] pointed out that on the PVP CE-CT images of patients with CRC, the filtered texture parameters of liver parenchymal were correlated with liver perfusion index and survival time; these findings provided a theoretical basis for the application of radiomics to analyze the heterogeneity of whole-liver parenchyma and further predict the prognosis of patients with CRC. Subsequently, an increasing number of studies found that when the liver parenchyma was affected by metastatic diseases, its heterogeneity can be reflected in changes in the CT texture characteristics of the liver. Rao et al. [25] reported that there were differences in texture parameters between different metastatic groups, and the AUCs for entropy and uniformity of liver parenchyma for diagnosing synchronous LM ranged from 0.73 to 0.78. Beckers et al. [26] verified that the uniformity difference between non-LM and simultaneous LM groups was statistically significant. Devoto et al. [27] also pointed out that there were differences in liver parenchyma between CRC patients with and without LM, and the liver parenchyma of patients with LM had higher levels of heterogeneous. In addition, other studies based on MR and unenhanced CT revealed the value of imaging features in predicting LM [28, 29]. This change in the texture characteristics of the liver may be due to the presence of micrometastases or occult metastases, resulting in abnormal blood perfusion. Although most of the existing studies on liver parenchyma analyzed a few common intensity features using simple statistical methods, their results have limited value. Some studies [30, 31] did not even find any significant feature that could predict LM. However, our study extracted 16 radiomics features most related to MLM from 1316 radiomics features and established prediction models showing higher clinical value than previous studies.

Taghavi et al. [32] retrospectively analyzed PVP CE-CT data in 91 cases of CRC. In their study, the clinical, radiomics, and combined models yielded AUCs of 71%, 86%, and 86% in the validation cohort. Our study achieved similar results. However, in our study, all patients with RC were treated surgically to eliminate the influence of the treatment scheme on the MLM as much as possible. In addition, Lee et al. [33] analyzed a part of segment 7 of the liver, and their results suggested that liver CT features had the potential to predict LM and could be used as an index to predict MLM. In our study, the VOI included the entire liver, which ensured more comprehensive feature coverage and yielded higher accuracy and repeatability. However, the accuracy and repeatability of liver segmentation were still associated with challenges that need to be further studied and unified. We applied only a manual segmentation method in our research. In the future, semi-automatic and automatic methods could be further compared and improved.

The models constructed in this study were helpful in predicting MLM within 24 months after RC surgery. Early prediction and detection of MLM can help patients at high risk of LM benefit from simultaneous resection or other adjuvant treatment. Other clinical interventions, including adjustment of the follow-up interval and the use of additional liver MR examinations, can also be performed to improve prognosis and survival [34].

Many risk factors, including clinical characteristics, postoperative pathological features, and genetic mutations, have been reported to relate to colorectal metastasis in previous studies [35–37]. However, there is no consensus regarding this issue. Our study analyzed the clinical and postoperative pathological features of these patients. Multivariate analysis showed that only the pT stage was an independent factor influencing MLM. However, the clinical model was less effective in predicting MLM (AUC of the validation set, 0.58) than the other two models. Moreover, the diagnostic efficiency of the combined model (AUC = 0.85) that combined pT stage and radiomics features was not significantly better than that of the radiomics model alone (AUC = 0.84). The pT stage could not play a role in preoperative prediction because it was evaluated on the basis of postoperative pathological findings. In this study, only the preoperative radiomics model achieved a good predictive effect. Moreover, clinical features showed limited value in this study. In the future, we will further explore the predictive value of clinical features and genetic information for MLM by expanding the sample size.

The postoperative treatment plans used in this retrospective study included some confounding factors. Some patients in the MLM group had indications for postoperative adjuvant treatment. However, for various reasons (for example, LM appeared soon after surgery or because patients did not receive adjuvant treatment in time due to a weak physique), some patients showed LM without timely adjuvant treatment, which changed the established treatment plans. In particular, patients who showed MLM soon after surgery often had liver micrometastases that could not be seen by the naked eye or shown by CT before the rectal operation. Thus, for patients misdiagnosed as having no LM before treatment, preoperative diagnosis was more important and may have changed their treatment plans. Therefore, the influence of postoperative adjuvant therapy on the occurrence of MLM in this study was unknown. Prospective research with a more standardized treatment plan in the future may provide more accurate information on the factors interfering with MLM.

This study had the following limitations. Firstly, this was a retrospective, single-center study that used different CT scanners. Prospective studies with external validation will be conducted in the future. Secondly, only PVP CE-CT was included in this study, it is the most widely used standardized follow-up protocol for RC patients. Third, although our study included relatively long-term follow-up, the false-positive predictions for patients whose MLM fell outside the scope of the follow-up period may have been overestimated. Finally, the accuracy and stability of liver segmentation need to be further studied and improved.

## Conclusion

In conclusion, a radiomics model based on preoperative whole-liver PVP CE-CT showed high value in predicting MLM within 24 months after RC surgery. The addition of clinical features failed to improve the prediction efficiency of the radiomics model.

## Supplementary Information

**Additional file 1.**

## Data Availability

The datasets analysed during the current study are available from the. corresponding author on reasonable request.

## Abbreviations

CRC: Colorectal cancer
LM: Liver metastases
MLM: Metachronous liver metastases
PVP: Portal venous phase
CE-CT: Contrast-enhanced CT
RC: Rectal cancer
pT: Pathological primary tumor
pN: Pathological regional lymph node
CA19–9: Carbohydrate antigen 19–9
CEA: Carcinoembryonic antigen
TME: Total mesorectal excision
VOI: Volume of interest
ICC: Interclass correlation coefficient
mRMR: Max-relevance and min-redundancy
LASSO: Least absolute shrinkage and selection operator
ROC: Receiver operating characteristic
AUC: Area under the curve
DCA: Decision curve analysis
OR: Odds ratio
CI: Confidence interval
