# Supplementary material for: Whole-liver enhanced CT radiomics analysis to predict metachronous liver metastases after rectal cancer surgery
Source: Cancer Imaging. 2022 Sep 11;22:50. doi: 10.1186/s40644-022-00485-z (PMC9465956; doi:10.1186/s40644-022-00485-z)
Supplement: Supplementary file 1 — Additional file 1. [file 40644_2022_485_MOESM1_ESM.pdf]

Supplementary Fig 1 Radiomic feature selection using LASSO method. Quantitative imaging texture features were extracted and selected to construct the radiomics model. The LASSO included choosing the regular parameter λ and determining the number of the features.

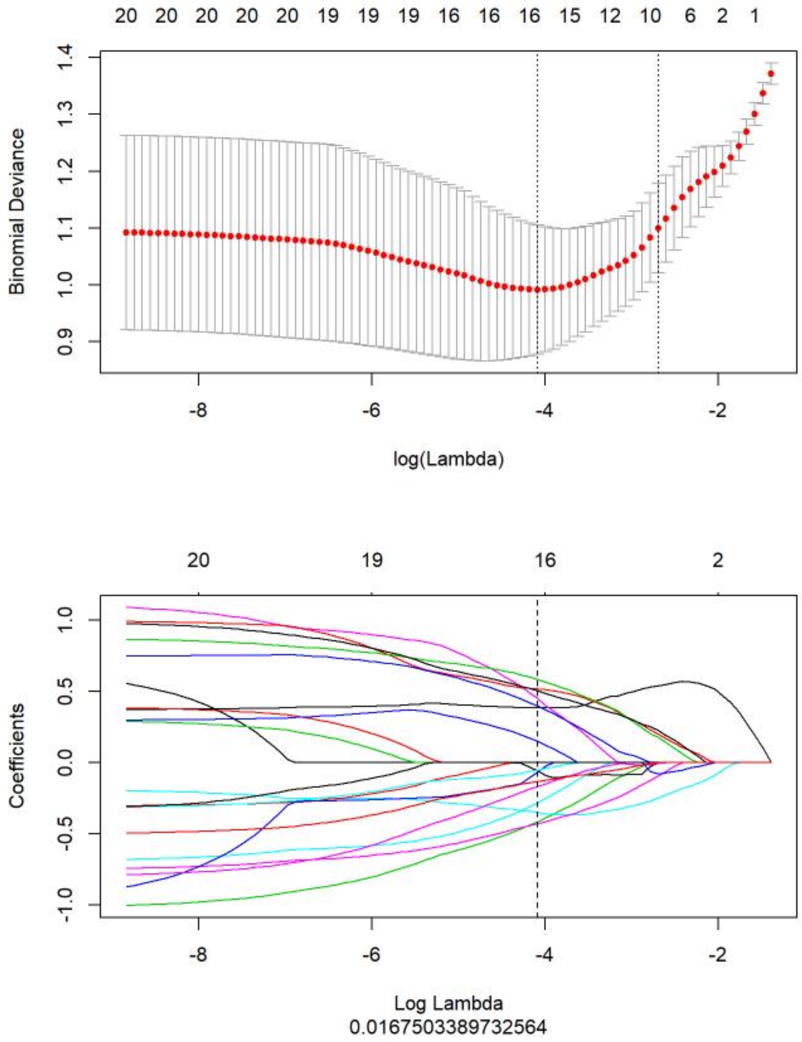

Supplementary Fig 2 LASSO-coefficients of the 16 selected radiomic features.

After dimensionality reduction by mRMR and LASSO, 16 radiomics features were finally selected from CE-CT images to construct a radiomics signature. The blue bar indicated the weight value of the radiomics features.

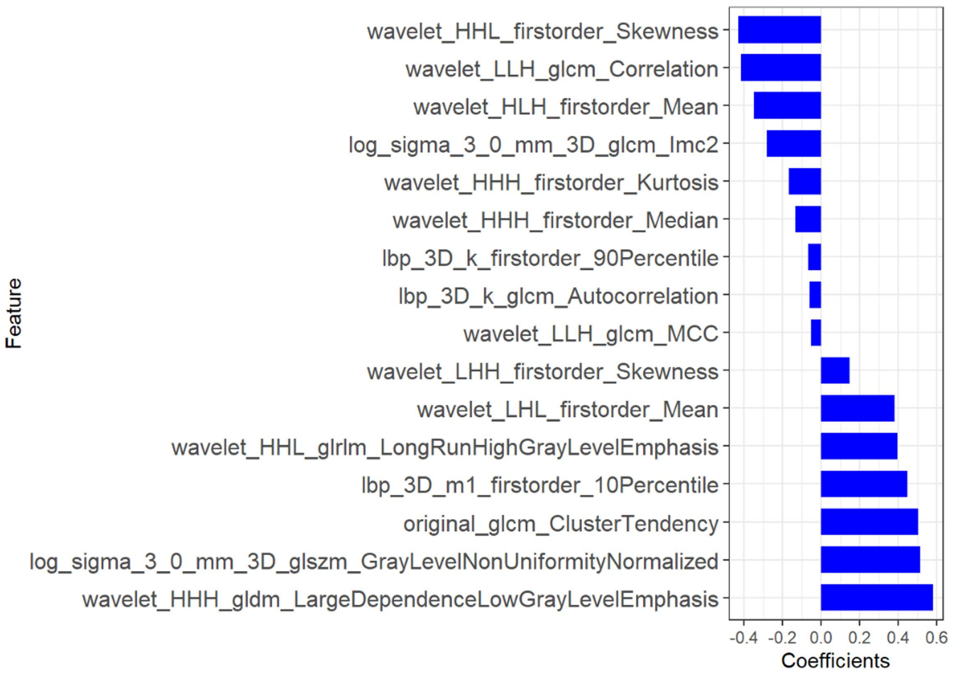

Supplementary Fig 3 The calibration curves of nomogram in the training and validation sets. The calibration curves revealed that the prediction probability of the nomogram was consistent with the actual event probability in the training set (A) and the validation set (B). Calibration curves depict the calibration of each model in terms of the agreement between the predicted event probability observed outcomes. The x-axis represents the predicted event probability and the y-axis the observed event probability. The diagonal gray line represents a perfect prediction by an ideal model. The pink punctate line represents the performance of the nomogram, of which a closer fit to the diagonal line represents a better prediction.

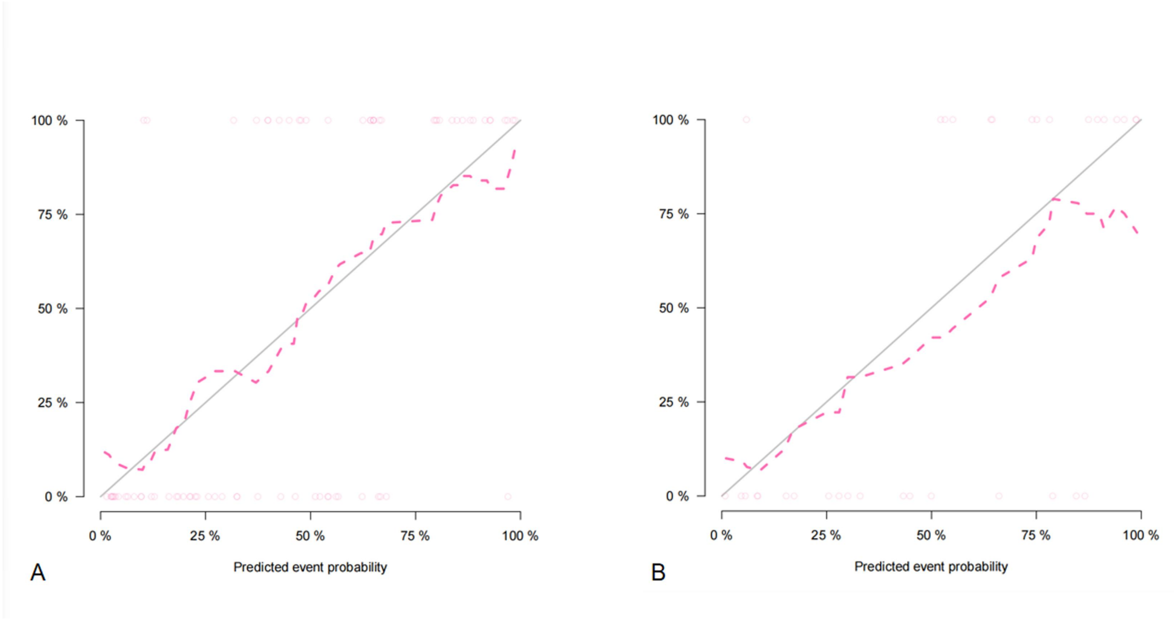

Supplementary formula

Radscore =

0.382*wavelet_LHL_firstorder_Mean+-0.416*wavelet_LLH_glcm_Correlation+0.147*wavelet_LHH_firstorder_Skewness+-0.351*wavelet_HLH_firstorder_Mean+0.447*lbp_3D_m1_firstorder_10Percentile+-0.061*lbp_3D_k_glcm_Autocorrelation+0.515*log_sigma_3_0_mm_3D_glszm_GrayLevelNonUniformityNormalized+0.581*wavelet_HHH_gldm_LargeDependenceLowGrayLevelEmphasis+0.397*wavelet_HHL_glrlm_LongRunHighGrayLevelEmphasis+-0.283*log_sigma_3_0_mm_3D_glcm_Imc2+-0.43*wavelet_HHL_firstorder_Skewness+0.502*original_glcm_ClusterTendency+-0.07*lbp_3D_k_firstorder_90Percentile+-0.054*wavelet_LLH_glcm_MCC+-0.17*wavelet_HHH_firstorder_Kurtosis+-0.134*wavelet_HHH_firstorder_Median + -0.216
